# SARS-CoV-2 Alchemy: Understanding the dynamics of age, vaccination, and geography in the evolution of SARS-CoV-2 in India

**DOI:** 10.1371/journal.pntd.0012918

**Published:** 2025-03-10

**Authors:** Mansi Patel, Uzma Shamim, Umang Umang, Rajesh Pandey, Jitendra Narayan

**Affiliations:** 1 CSIR Institute of Genomics and Integrative Biology (CSIR-IGIB), New Delhi, India; 2 Academy of Scientific and Innovative Research (AcSIR), Ghaziabad, India; Zhejiang Wanli University, CHINA

## Abstract

**Background:**

COVID-19 pandemic had unprecedented global impact on health and society, highlighting the need for a detailed understanding of SARS-CoV-2 evolution in response to host and environmental factors. This study investigates the evolution of SARS-CoV-2 via mutation dynamics, focusing on distinct age cohorts, geographical location, and vaccination status within the Indian population, one of the nations most affected by COVID-19.

**Methodology:**

Comprehensive dataset, across diverse time points during the Alpha, Delta, and Omicron variant waves, captured essential phases of the pandemic’s footprint in India. By leveraging genomic data from Global Initiative on Sharing Avian Influenza Data (GISAID), we examined the substitution mutation landscape of SARS-CoV-2 in three demographic segments: children (1–17 years), working-age adults (18–64 years), and elderly individuals (65+ years). A balanced dataset of 69,975 samples was used for the study, comprising 23,325 samples from each group. This design ensured high statistical power, as confirmed by power analysis. We employed bioinformatics and statistical analyses, to explore genetic diversity patterns and substitution frequencies across the age groups.

**Principal findings:**

The working-age group exhibited a notably high frequency of unique substitutions, suggesting that immune pressures within highly interactive populations may accelerate viral adaptation. Geographic analysis emphasizes notable regional variation in substitution rates, potentially driven by population density and local transmission dynamics, while regions with more homogeneous strain circulation show relatively lower substitution rates. The analysis also revealed a significant surge in unique substitutions across all age groups during the vaccination period, with substitution rates remaining elevated even after widespread vaccination, compared to pre-vaccination levels. This trend supports the virus's adaptive response to heightened immune pressures from vaccination, as observed through the increased prevalence of substitutions in important regions of SARS-CoV-2 genome like ORF1ab and Spike, potentially contributing to immune escape and transmissibility.

**Conclusion:**

Our findings affirm the importance of continuous surveillance on viral evolution, particularly in countries with high transmission rates. This research provides insights for anticipating future viral outbreaks and refining pandemic preparedness strategies, thus enhancing our capacity for proactive global health responses.

## Author summary

The COVID-19 pandemic, showed surge of different variants of SARS-CoV-2 across different time points, affecting millions globally and highlighting the importance of understanding how viruses change over time. This study explored how SARS-CoV-2 evolved in India, particularly focusing on the role of age, location, and vaccination in driving substitution mutations. By analyzing the genome data of the virus collected in India from different age groups—children, working-age adults, and the elderly—we found that working-age adults showed a high number of unique substitutions. This suggests that stronger immune responses in this age group may drive the virus to adapt more quickly. We also discovered that during the vaccination period, substitution rates increased across all age groups, with these rates staying high even after widespread vaccination. This may mean that both natural and vaccine-induced immunity can encourage the virus to mutate. Our findings suggest that local conditions, like population immunity and vaccination coverage, can shape the virus's substitution patterns. Continuous monitoring of these changes is essential for preparing for future pandemics, especially in high-risk areas like India, helping us improve global health responses and prevent new outbreaks.

## Introduction

**Fig 1:**
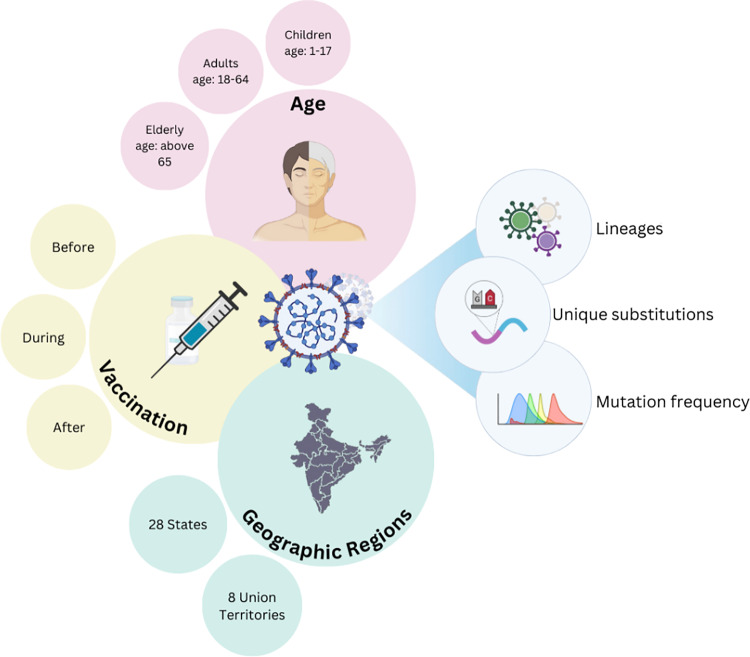
Image illustrates the key factors discussed in this study that may influence viral evolution, including different age, geographic regions of India, and vaccination efforts. Figure created with licensed version of Biorender.com.

The COVID-19 pandemic, a global outbreak that emerged in late 2019, has had a significant and extensive influence on nearly every aspect of human society. The virus has spread around the world in the five years since the first case was recorded on December 31, 2019 [1]. COVID-19 pandemic has officially ended, but this is not the first time a viral pathogen has caused severe harm to human health and the global economy. Historical examples, such as the 1918 influenza pandemic (H1N1), which resulted in an estimated 50 million deaths worldwide [2], and the 2009 H1N1 influenza outbreak led to an estimated 151,700–575,400 deaths [3], highlight the recurring threat of viral evolution. Globally, 80 percent of (H1N1) pdm09 virus-related deaths were estimated to have occurred in people younger than 65 years of age. This differs greatly from typical seasonal influenza epidemics, during which about 70 percent to 90 percent of deaths are estimated to occur in people 65 years and older [4]. Age, therefore, can play a significant role in how a virus evolves and adapts within the host, as different age groups may present unique immunological pressures that shape viral mutations and transmission dynamics. The ongoing evolution of SARS-CoV-2 [5], as well as other viruses like influenza [6] and HIV [7], underscores the importance of continuous surveillance and research into viral behavior. Understanding the mechanisms of viral evolution, through mutations and selective pressure, is critical to preparing for and mitigating the impact of future pandemics [8–10]. Immunocompromised individuals, such as those with chronic illnesses, advanced age, or on immunosuppressive treatments, may struggle to eliminate the virus as quickly as healthy individuals [11], creating ideal conditions for mutations to arise [12,13]. This extended period of viral replication allows more opportunities for mutations to occur [14]. However, viral evolution is not limited to immunocompromised hosts. Even in healthy and younger individuals, the virus continues to evolve [12]. In these hosts, the immune system typically responds more effectively, clearing the infection more quickly. Despite the swift immune response, high transmission rates among younger populations [15], coupled with their often mild or asymptomatic infections, can create opportunities for the virus to spread unnoticed. It is also linked to metabolic abnormalities like insulin resistance and altered glucose metabolism, which can worsen conditions such as obesity and type 2 diabetes [16]. Multiple strains have now emerged, some of which exhibit increased transmissibility [17,18]. Given the complex and varied nature of viral evolution, research into viral adaptation is crucial. India is among the most affected nations by SARS-CoV-2 [19]; the surveillance is essential to understand the dynamics of the virus evolution in the Indian population. This study provides critical insights into how age-related factors influence SARS-CoV-2 evolution; by revealing patterns of genetic diversity among different age cohorts. The objective of this study is to investigate the evolutionary dynamics of SARS-CoV-2 in India by analyzing genomic variations across the viral samples with a specific focus on differences related to age groups, geographic regions, and the distinct phases of vaccination. Leveraging genomic data from India through the Global Initiative on Sharing Avian Influenza Data [20] (GISAID) database, this research focuses on the surveillance of SARS-CoV-2 substitution mutations across different age cohorts within the Indian population. This study hypothesizes that age-related differences in immune responses and geographic variations in transmission dynamics significantly may influence SARS-CoV-2 mutation patterns. Children, working-age adults, and the elderly present distinct immunological environments, with varying exposure levels and immune pressures driving unique substitution rates. Geographic factors such as population density and healthcare access further shape regional mutation dynamics. By focusing on these demographic and geographic factors within India’s diverse population, the study aims to elucidate critical drivers of viral evolution. Utilizing bioinformatics tools and statical analysis techniques, this study investigates the landscape of substitutions within three distinct age groups: children (1-17 years), working-age adults (18-64 years), and elderly individuals (65 years and above) in India. The analysis reveals intriguing insights into the genetic diversity of SARS-CoV-2 within these demographic segments, shedding light on potential age-related patterns of viral evolution. A notable finding of this study is the discrepancy observed in the number of unique substitutions among different age groups, particularly within the working-age adult cohort in India. Moreover, we have also attempted to analyze the shift in the patterns of unique substitutions with reference to SARS-CoV-2 lineages captured across different time points, different geographic regions of India and vaccination status of the SARS-CoV-2 infected individuals, summarized in Fig 1.

## Methodology

### Data acquisition

The initial dataset, comprising SARS-CoV-2 genomes, was sourced from the Global Initiative on Sharing All Influenza Data (GISAID) database. As of August 28, 2023, this dataset included a total of 15,905,287 genome sequences from various countries worldwide. The comprehensive dataset provided a robust foundation for the analyses. For Indian region-specific analysis, 293,730 genome sequences from India were initially considered. To ensure the integrity and reliability of the data, only sequences with genome completeness exceeding 95% were included in the analysis. Sequences with genome lengths shorter than 27,000 base pairs (bp) were excluded to maintain quality standards. The completeness and quality of the selected sequences were assessed using the CheckV tool [21], which rigorously evaluates viral genome completeness. For age-group-specific SARS-CoV-2 variant surveillance, sequences lacking essential metadata such as age, sample collection date, and geographic information (region/state) were excluded. The final dataset included 219,149 sequences, which were categorized into three distinct age groups: children (aged 0-17), working-age individuals (aged 18-64), and elderly adults (aged 65 and above). The distribution of sequences was as follows: 23,325 for children, 171,363 for working-age individuals, and 24,461 for the elderly. To address the disparities in sample size across the age groups and ensure unbiased comparisons, an under sampling method was employed using the sample() function in R. This approach selectively reduced the larger groups to match the size of the smallest group, thereby minimizing potential bias due to the inadvertent and unavoidable unequal sample sizes. A total of 23,325 samples were randomly selected from each of the working-age, elderly, and children’s group, resulting in a balanced dataset comprising of 69,975 samples (23,325 per age group) as detailed in Table 1.

**Table 1:** Distribution of samples by age group and gender.

Age Group	Gender	Sample Count		
Children (1–17)	Female	10781	23325	69975
	Male	12544		
Working-age (18–64)	Female	9779	23325	
	Male	13546		
Elderly (65+)	Female	9480	23325	
	Male	13845		

Random subsampling also ensured equal temporal representation across months within all age groups. The Kolmogorov-Smirnov (KS) test was performed in R to compare the temporal distribution of sample collection dates across the three age groups using the ks.test() function, where the observed distribution was tested against a uniform distribution within the study period. The results are summarized in Table 2.

**Table 2:** Kolmogorov-Smirnov test results for temporal distribution.

Comparison	KS Test Statistic (D)	*p-Value*
Children vs Adults	0.11905	0.916
Children vs Elderly	0.095238	0.9904
Adults vs Elderly	0.11905	0.9203

**Fig 2:**
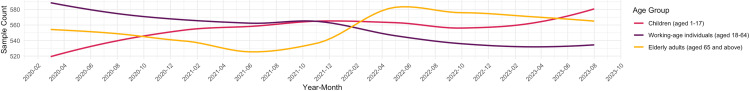
Temporal Distribution of Samples used in the study Across the Age Groups. It captures the time period between March 2020 to August 2023 of the COVID-19 cases in India.

The KS test results indicated no significant differences in the temporal distribution of samples across the three age groups, suggesting that the variation in sample counts over time is consistent across all groups. The p-values (greater than 0.05) support the null hypothesis that the distributions are similar. A time series plot (Fig 2) illustrated the monthly distribution of samples across the three age groups.

For geographic distribution, we preserved the original proportional distribution of samples across states to maintain authenticity. Variations in sample availability among states, often influenced by regional surveillance priorities, were addressed by proportionate subsampling based on the available data from each state.

To ensure the validity of our study, we performed a power analysis using the observed effect size (Cohen’s w = 0.0327), sample size (N = 69,975; 23,325 per group), and degrees of freedom (df = 2). The power analysis was conducted using the pwr.chisq.test() function from the pwr package in R. The results indicated a high statistical power of 1, indicating that the study is well-powered to detect significant differences across the age groups. This power ensures that the observed findings are reliable and not due to a potential Type II error. Additionally, vaccination status of the SARS-CoV-2 infected individuals were taken into account from GSIAID data and used to categorize the samples whether falling into time frames of before, during, or after the vaccination started in India. In India, the vaccination began on January 16, 2021, and achieved a milestone of over 1 billion vaccinations by November 2021 [22,23]. For our analysis, the “before vaccination” period included data prior to January 16, 2021; the “during vaccination” phase spanned from January 16, 2021, to November 30, 2021, reflecting the rapid rollout and coverage expansion; and the “after vaccination” phase included data post-November 2021, marking the point when vaccination rates plateaued, and booster campaigns were introduced. These timeframes aligned with key milestones in the vaccination timeline and provided a framework for assessing temporal trends.

### Variant Detection and Annotation

The C-Sibelia [24] tool was utilized for variant detection. This tool compares sample FASTA sequences to the reference genome, SARS-CoV-2 isolates Wuhan-Hu-1 (Genome assembly: ASM985889v3), and identifies variations. The output is a Variant Call Format (VCF) file containing all identified single nucleotide variations (SNVs) and indels. For this study, only SNVs were considered for this analysis due to their higher frequency in SARS-CoV-2 genomes and their significant role as key substitutions that affect viral transmissibility, immune evasion, and pathogenicity [25]. SNVs are more reliably detected in large-scale genomic datasets, minimizing potential errors associated with more complex variants like insertions and deletions (indels). By concentrating on SNVs, we enhance data accuracy, reduce computational complexity, and align our study with established SARS-CoV-2 research, allowing for a more focused and actionable analysis of variant patterns across different cohorts. To ensure robustness and reduce the likelihood of random occurrences, only substitutions occurring in five or more samples were included in the analysis. Variant annotation was carried out using the snpEff [26] tool, which enriches genomic variants with functional predictions. This tool categorizes the variants based on their phenotypic effects, providing insights into the potential impact of each substitution.

### Data Analysis

The annotated data was analyzed using programming language R (4.4.0). This enabled a comprehensive analysis of the genomic variants, facilitating the identification of patterns and trends. The R script utilized for the analysis is available at the following GitHub repository [https://githhub.com/siya-00SARS-CoV-2_Alchhemy].

### Metrics for Assessing Mutation Diversity

Chi-square tests evaluated the association between age groups and unique substitution patterns, with pairwise Chi-square tests, applying Bonferroni correction to control for multiple comparisons, conducted using the chisq.test() function in R.

The Friedman rank sum test was employed to evaluate differences in mutation frequencies across vaccination status, stratified by age groups. This non-parametric test was chosen due to its suitability for analyzing dependent groups, as it does not require the data to meet the assumptions of normality or homogeneity of variance. The test’s robustness to non-normal distributions ensures valid results in datasets where absolute values may vary but relative patterns are critical for interpretation. The test was implemented using the friedman.test() function in R.

The unique substitution ratio, calculated as the proportion of unique substitutions to total substitutions, provided a normalized measure of substitution diversity for each age group and region. This ratio facilitated meaningful comparisons across demographic and geographic variables, highlighting age-specific and regional differences in mutation dynamics. Similarly, the mutation/substitution frequency was calculated by dividing the total number of substitutions by the number of samples in each group, allowing for the evaluation of how substitution rates varied across different conditions, such as vaccination phases and age groups. This standardization enabled direct comparison of substitution rates across genes of varying sizes, presented as percentages to enhance clarity. To ensure accurate comparisons of substitution counts across genes of varying sizes, we standardized the counts based on the length of each gene in nucleotides, as obtained from SARS-CoV-2 genome annotations. This standardization accounts for gene size differences, allowing for direct comparison of substitution rates across genes. The resulting values are expressed as percentages to enhance clarity.

## Results

### Age-stratified analysis reveal unique substitution mutational patterns in working-age population

This study utilized a comprehensive dataset comprising 69,975 samples, with an equal distribution of 23,325 samples from each of the three age groups: children (1-17 years), working-age individuals (18-64 years), and elderly adults (65 years and above). For each group, the samples were taken from all 28 states and 8 union territories, ensuring geographical diversity across the Indian population. Slight male predominance was noted in all age groups, attributable to sample availability. The dataset spans a wide timeframe, from March 2020 to August 2023, capturing key periods of the pandemic in India. This timeframe aligns with the circulation of multiple SARS-CoV-2 variants, including Alpha, Delta, and Omicron. Given the random subsampling approach applied, the dataset contains a mix of lineages, ensuring that the substitution spectra corresponding to each major variant are well represented. The inclusion of varied demographics and geographic regions enhances the robustness of the analysis, ensuring that it reflects the diverse impact of SARS-CoV-2 across different population segments in India. This approach also accounts for potential variations in viral evolution due to local epidemiological factors and provides a broad view of genomic dynamics in different groups. Focusing on substitutions present in five or more samples to ensure that only consistently observed substitutions, rather than isolated or random occurrences, are included in the analysis. The analysis revealed a total of 13,882 substitutions in children, 15,038 substitutions in the working-age cohort, and 14,373 substitutions in the elderly population. Among the total substitutions observed across all groups, we specifically identified those that were present in only one group and absent in the others. These substitutions were classified as unique to that particular group, suggesting potential group-specific genetic variations. Notably, the working-age segment displayed the highest count of unique substitutions. In the pediatric cohort, we identified a total of 4,240 unique substitutions, of which 2,482 were classified as synonymous whilst 1,758 were nonsynonymous substitutions, where synonymous accounted for 58.7% of the total unique substitutions. Interestingly, the working-age population displayed a higher count of unique substitutions, totaling 5,429 with 3,188 categorized as synonymous and 2,241 as nonsynonymous substitutions. The elderly cohort exhibited 4,697 unique substitutions, among which 2,735 were synonymous and 1,962 nonsynonymous substitutions. The working and elderly cohort exhibited similar percentages of synonymous substitutions (58.7% and 58.2% respectively) to the pediatric cohort.

We used a Chi-squared test to evaluate differences in unique substitution counts among the three groups. The analysis compared the observed and expected counts of unique substitutions across three age groups: Children (aged 1-17), Working-age individuals (aged 18-64), and Elderly adults (aged 65 and above). The observed counts of substitutions in these groups were 4240, 5429, and 4697, respectively. For each group, the expected number of substitutions, assuming no differences between the groups, was 4788.67. The Chi-square statistic for each group was calculated by the formula (O−E)2/E(O - E)^2/ E(O−E)2/E, where “O” is the observed count and “E” is the expected count. The contributions to the Chi-square statistic were 60.734 for children, 41.037 for working-age individuals, and 48.469 for elderly adults, giving a total Chi-square statistic of 150.24. With 2 degrees of freedom, the resulting p-value was less than 2.2e-16, indicating a statistically significant difference between the observed and expected substitutions counts across the age groups. To further strengthen the findings from the Chi-squared test, we performed pairwise Chi-squared tests to compare the number of unique substitutions between groups individually. To control for the risk of type I errors due to multiple comparisons, we applied the Bonferroni correction to the p-values obtained from these tests. Table 3 presents pairwise comparisons of observed counts, p-values and adjusted p-values. The statistical analyses performed, including both the initial Chi-squared test and the subsequent pairwise comparisons with Bonferroni correction, consistently indicate that working adults possess a significantly higher number of unique substitutions compared to other groups. This finding is crucial as it suggests that the higher unique substitution count in working adults is not merely due to random variation but represents a significant difference.

**Table 3:** Pairwise comparisons of age groups using chi-square statistics.

Comparison	Observed (O)	p-value	Adjusted p-value
Children (1–17) vs. Working-age (18–64)	4240, 5429	< 2.2E- 16	3.50E-33
Working-age (18–64) vs. Elderly (65+)	5429, 4697	3.48E- 13	1.04E-12
Children (1–17) vs. Elderly (65+)	4240, 4697	1.34E- 06	4.01E-06

# The observed counts correspond to the number of unique substitutions in each group.

The results confirm that working-age individuals (18–64) have a significantly higher number of unique substitutions compared to children (1–17) and elderly adults (65 and above), as shown in Table 1. Statistical analyses, including the Chi-squared test for independence and pairwise comparisons with Bonferroni correction, consistently support this finding. This significant difference underscores a distinct substitution profile in the working-age group, suggesting a non-random pattern in substitution prevalence.

### Lineage dynamics and demographic influences on unique substitutions

**Fig 3:**
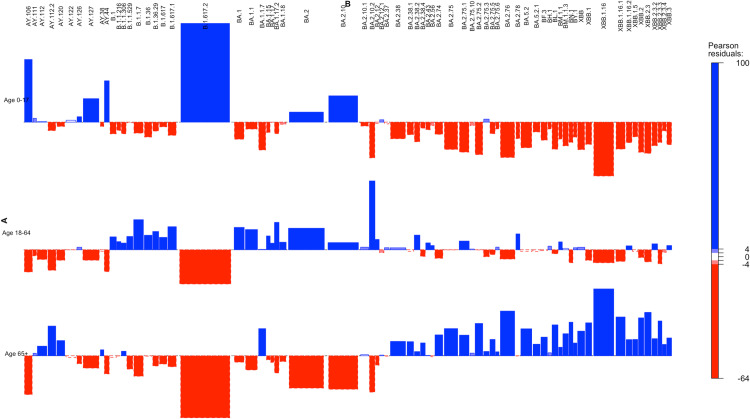
The plot illustrates the association Between SARS-CoV-2 Lineages and Age Groups Based on Observed Number of Substitutions. The x-axis represents the different viral lineages, while the y-axis, transformed logarithmically, displays the count of unique substitutions associated with each lineage.

Variation in unique substitutions likely reflects differences in the circulation of SARS-CoV-2 variants across age groups, influenced by underlying biological or environmental factors warranting further investigation. We analysed the lineages associated with unique substitutions to identify patterns in lineage prevalence and unique substitutions across different age groups. Viral lineage distribution typically follows a power-law pattern, with a few dominant lineages and many rare ones. To investigate the relationship between SARS-CoV-2 lineages and age groups, we performed a Chi-squared association analysis using a contingency table that captured the count of substitutions within each lineage across three age categories: children (0–17 years), working-age (18–64 years), and elderly (65+ years). The analysis revealed a significant association between the number of substitutions present in lineages and age groups (X² = 94,672, df = 140, p < 2.2e-16), confirming a strong dependency between these variables. The association plot (Fig 3) illustrates distinct patterns in lineage prevalence across the age groups, with Delta-associated lineages (e.g., AY.120, AY.38, AY.39, AY.106) disproportionately observed in children and elderly groups, whereas Omicron variants (e.g., BA.1.1, BA.2) were predominantly associated with the working-age population.

The analysis reveals a distinct divergence in the substitution load across different age groups for Delta and Omicron variants (Fig 3). For lineages associated with the Delta variant, such as AY.120, AY.38, AY.39, AY.1, AY.106, AY.4, AY.44, and AY.98, the elderly and pediatric groups exhibit a higher number of unique substitutions compared to the working-age group. Similar to these observations, studies utilizing data from Delhi highlighted the Delta variant’s role in breakthrough infections during the second wave [27]. Despite moderate vaccination coverage, the Delta variant’s partial immune escape contributed to reinfections and reduced vaccine effectiveness [27]. In contrast, Omicron variants like BA.1.1 and BA.2 show higher unique substitution counts in the working-age group. This observation aligns with the increased transmissibility associated with Omicron variants [28]. Omicron has been identified as more transmissible than Delta, largely due to a higher number of substitutions in its spike protein, several of which enhance its ability for rapid transmission [29]. The predominance of Omicron in the working-age population likely correlates with this age group’s higher viral exposure and transmission dynamics due to increased social interactions and mobility.

**Fig 4:**
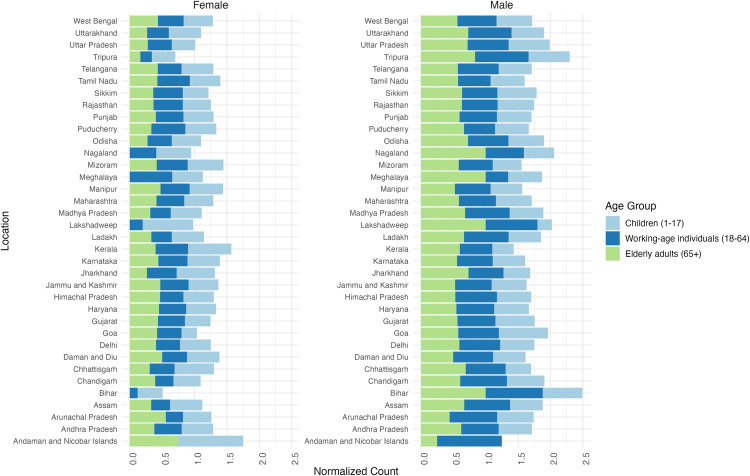
Distribution of normalized count of unique substitutions across different age groups and genders in various Indian states. Bar plots representing this distribution is summarized in this figure.

**Fig 5:**
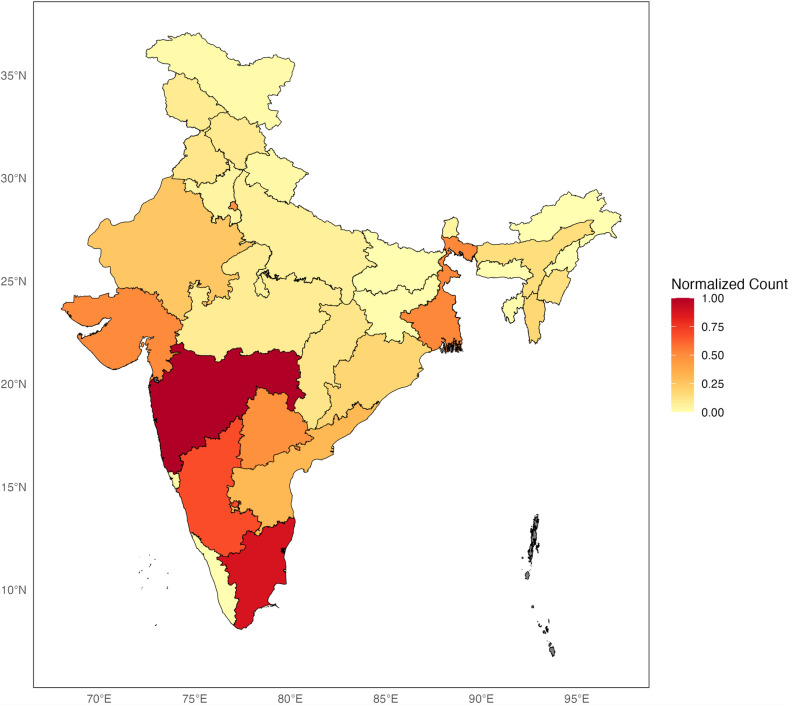
Map illustrates the distribution of normalized count of unique substitutions across Indian states. Each state color is based on the number of unique substitutions found within its population. This figure is created using shapefile data sourced from DataMeet GitHub repository: [https://github.com/datameet/maps/tree/master/States]. Shapefile data is licensed under MIT License (2020).

We analyzed the distribution of unique SARS-CoV-2 substitutions across different demographic groups (age and gender) and geographic regions in India, as depicted in Figs 4 and 5. Understanding gender-specific substitution trends help unravel any potential biological or social factors contributing to differential viral behaviour between males and females. The geographic analysis highlights regional disparities in substitution frequency, shedding light on how local factors, such as population density or public health interventions, may influence viral diversity.

While the number of unique substitutions varies significantly between states, a general trend emerges: Although unique substitution counts vary between states, working-age individuals consistently show higher substitution frequencies*.* Additionally, the distribution of substitutions between genders shows some variation across states, with certain states displaying a higher prevalence of substitutions in males and others in females. Overall, the visualization highlights the complex interplay between age, gender, and geographic location in shaping the genetic landscape of SARS-CoV-2 in India’s population.

Similarly, Fig 5 illustrates the distribution of unique substitutions across the country. While some states in the central and southern regions exhibit notably high number of unique substitutions, represented by darker shades of red, other states, particularly in the north and northeast, display lower counts indicated by lighter colours. This visualization underscores the heterogeneous genetic landscape across India and highlights potential regional disparities in genetic diversity of SARS-CoV-2 among adults.

### Comparative analysis of unique substitution ratio for age groups reveals significantly elevated rates in working-age individuals across majority of states in India

This analysis examines unique substitution ratio across various states in India to provide insights into the regional and age-related dynamics of SARS-CoV-2 substitutions. The unique substitution ratio, calculated as the proportion of unique to total mutations/substitutions, was analysed across Indian states and age groups, revealing the rate at which these substitutions have occurred over time. Higher percentage can indicate a greater likelihood of beneficial substitutions arising, which are essential for adaptation and evolution.

**Fig 6:**
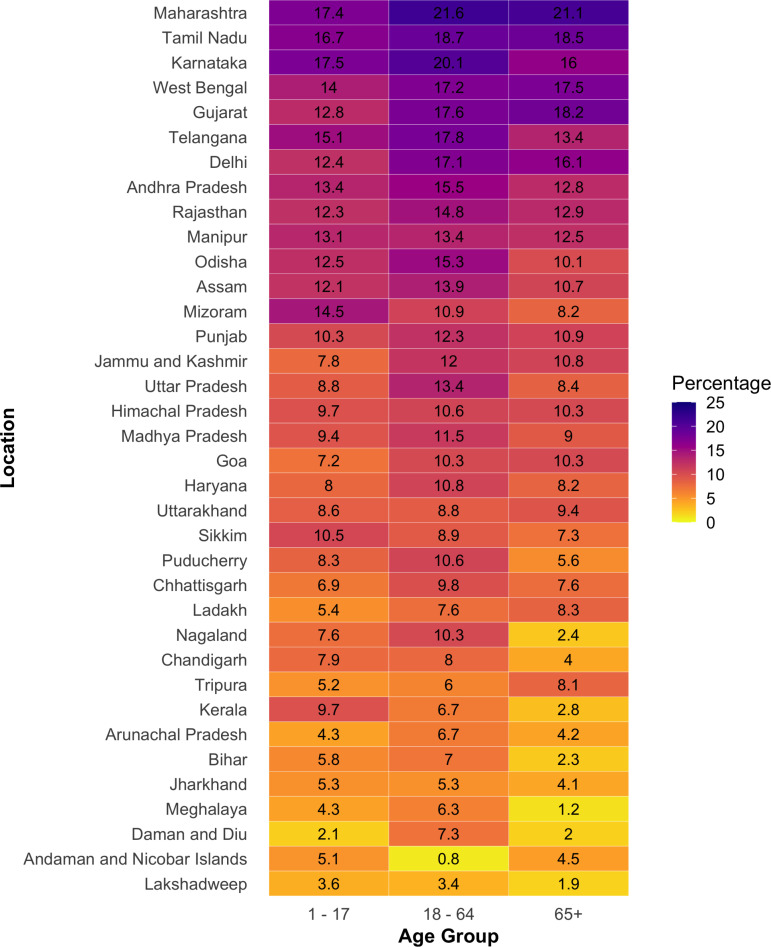
Heatmap illustrating the distribution of unique substitution ratio across different age groups and Indian states. Each state is represented by a row, and the columns correspond to age groups: children (1–17), working-age adults (18–64), and elderly (65+).

This analysis of SARS-CoV-2 unique substitution ratio across various age groups in India reveals a notable pattern, with average of 9.60% for children (1-17 years), 9.22% for the elderly (65+ years), and 11.34% for the working-age group (18-64 years) (Fig 6). This pattern indicates that working-age individuals generally exhibit a higher rate of unique substitutions among total substitutions than both children and elderly adults. Some significant regional variations are evident. States such as Maharashtra and Karnataka exhibit the highest percentage, with rates reaching more than 20% for working-age individuals. This high in certain regions may point to the circulation of specific viral strains or differences in local viral transmission dynamics. Conversely, states such as Kerala and Mizoram exhibit lower percentage, with higher rates observed in children and elderly individuals compared to working-age individuals. These lower rates could be indicative of a more homogeneous viral strain presence or possibly less aggressive viral strains in those areas. A notable pattern is the relatively high diversity percentage in some states, which could be attributed to several factors including regional variations in public health interventions, differences in the effectiveness of vaccination campaigns, or variations in virus strain diversity. For instance, states with high substitution rates might be experiencing more frequent viral substitutions due to a combination of higher transmission rates and a broader range of circulating variants.

### COVID-19 vaccination linked to higher substitution frequencies in India’s elderly

To analyze the impact of COVID-19 vaccination on substitution frequency in India, the substitution frequency is calculated by counting the total number of substitutions for each age group, location, and vaccination status, then dividing this count by the number of samples in that group. For unique substitution frequency, only the count of unique substitutions is considered, instead of the total number of substitutions. This provides a normalized measure of substitution frequency, allowing for comparisons across different age groups and vaccination periods. We examined data across various states and age groups before, during, and after the initiation of the vaccination campaign, which began on January 16, 2021 and over 1 billion people were vaccinated by November 2021 [22,23]. By comparing substitution frequencies recorded before, during, and after the vaccination rollout, we aimed to determine whether vaccination had any discernible effect on these metrics. Recent research has examined the role of vaccination in the emergence of new variants of SARS-CoV-2. The findings indicate that while vaccination reduced the infection rate of SARS-CoV-2 in Indian population, yet, vaccination breakthrough cases did occur highlighting the probable role of vaccination in the development of immune-evading strains when transmission rates within the population remained high. This suggested that, although various variants have emerged at different times, vaccination breakthrough has influenced the overall emergence of new variants, likely contributing to an increased number of substitutions [30]. Further discussion in another study highlights that mass vaccination may accelerate SARS-CoV-2 evolution, particularly in antibody-binding regions, compared to natural infection [31]. While vaccines play a crucial role in significantly reducing hospitalizations and deaths, they may also create selection pressure on the virus, facilitating substitutions that promote immune escape. While it is true that the observed increase in substitution rates during, and after vaccination can be attributed to the circulation of various SARS-CoV-2 strains such as Delta, and Omicron; it is essential to recognize that vaccination still plays a significant role in shaping these substitution dynamics. Although different variants have emerged at different times, the vaccination rollout has directly influenced substitution rates by altering the selective pressures on the virus. Despite high levels of natural and vaccine-induced immunity, breakthrough infections have been observed, often associated with VOCs. For instance, documented cases of breakthrough reinfections involving Alpha and Delta variants illustrate the capability of these VOCs to bypass existing immunity, leading to severe outcomes even in individuals with robust immune responses [32].

**Fig 7:**
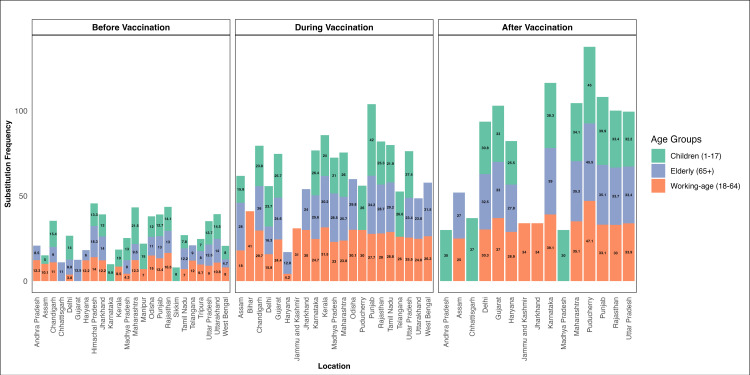
The bar plot illustrates the substitution frequency across various locations in India, segmented by three different time periods: before vaccination, during vaccination, and after vaccination. The data is further divided into three age groups: Children (1-17 years), Elderly (65+ years), and Working-age individuals (18-64 years), represented by green, blue, and orange bars, respectively. Each location shows the cumulative substitution frequency, with distinct patterns observed in different periods. This visual representation underscores the variations in substitution frequency by age group and location, influenced by the vaccination timeline.

The bar plot (Fig 7) shows variation in substitution frequency across Indian locations and vaccination periods, with notable increases during the vaccination phase among the working-age group and post-vaccination among children and the elderly. This underscores the impact of vaccination phases and demographics on substitution frequency trends.

Our data revealed a general trend, during the vaccination period, substitution frequencies appear relatively high across many locations, particularly for the working-age group. We observed increased substitution frequencies in many states post-vaccination. For instance, states like Delhi and Karnataka exhibited higher substitution frequencies after vaccination compared to the pre-vaccination phase. While vaccination primarily exerts selection pressure on the SARS-CoV-2 spike protein, our analysis includes substitutions observed across the entire viral genome to capture the broader evolutionary dynamics. This approach reflects the complex interplay of direct and indirect effects that shape the virus’s adaptation to both immune responses and other environmental factors. This suggests that vaccination campaigns, while crucial for controlling the virus, might be associated with an increase in detected substitutions. However, the extent of this increase varied significantly across different regions. Age-specific patterns were also observed. In the children (1–17) group, substitution frequencies varied widely, with some states like Delhi and Karnataka showing substantial increases during the vaccination period, while others like Kerala and Madhya Pradesh exhibited more stable substitution frequencies. The elderly (65+) group generally displayed higher substitution frequencies during vaccination. The working-age (18–64) group also showed varied trends, with some states experiencing increased frequencies post-vaccination and others showing decreases. Overall, while many states experienced elevated substitution frequencies during and after the vaccination period, notable exceptions existed. States like West Bengal and Odisha reported lower substitution frequencies, which could be attributed to differences in healthcare infrastructure, testing practices, or infection rates.

The Friedman test offers a statistically rigorous approach to identifying temporal trends in substitution frequency for different age groups. This analysis revealed significant differences in substitution frequencies across vaccination phases when stratified by age groups with p value 0.04. These results indicate that the mutation frequencies varied significantly across the three vaccination periods: before vaccination, during vaccination, and after vaccination. The highest mutation frequencies were observed during the vaccination. This observation underscores the temporal impact of vaccination campaigns on viral evolution.

### Observations on unique substitution counts across vaccination phases and age groups

The analysis of unique substitutions across different age groups and vaccination phases reveals significant variations in substitution dynamics. For the children (1–17) age group, there is a notable increase in unique substitutions from the pre-vaccination phase to during-vaccination. Specifically, unique substitutions rose from 596 before vaccination to 1401 during vaccination, suggesting a substantial increase in substitution diversity as vaccination efforts intensified. The count of unique substitutions after vaccination decreased to 883, indicating a reduction compared to the peak during vaccination but still higher than pre-vaccination levels.

The elderly (65+) age group exhibits a similar trend. Unique substitution counts increased from 307 before vaccination to 1394 during vaccination, highlighting a significant rise in substitution diversity during the vaccination campaign. After vaccination, the count reduced to 823, reflecting a decrease from the peak period but still demonstrating a considerable increase compared to the pre-vaccination period. This pattern suggests that while vaccination may have led to a surge in unique substitutions, the effect diminished somewhat after the vaccination period.

The working-age (18–64) group shows the most pronounced increase in unique substitutions across all phases. The count surged from 345 before vaccination to 1595 during vaccination, indicating the highest level of substitution diversity during this period. Post-vaccination, the unique substitution count was 1191, which, while lower than during vaccination, remains substantially higher than pre-vaccination levels. This trend suggests that the working-age group experienced a significant rise in unique substitutions as a result of the vaccination campaign. The vaccination campaign, while pivotal in reducing severe infections, exerted selective pressures on SARS-CoV-2. The Delta variant demonstrated reduced sensitivity to vaccine-induced antibodies and was associated with breakthrough infections, particularly among ChAdOx1 vaccinees [33]. Breakthrough infections (VBT) in healthcare workers (HCWs) after ChAdOx1 nCoV-19 vaccination, predominantly driven by the Delta variant, have highlighted the challenges posed by these VOC. Fully vaccinated individuals demonstrated higher protection and humoral immune responses compared to partially vaccinated or unvaccinated individuals, although breakthrough cases were still observed [34].

**Fig 8:**
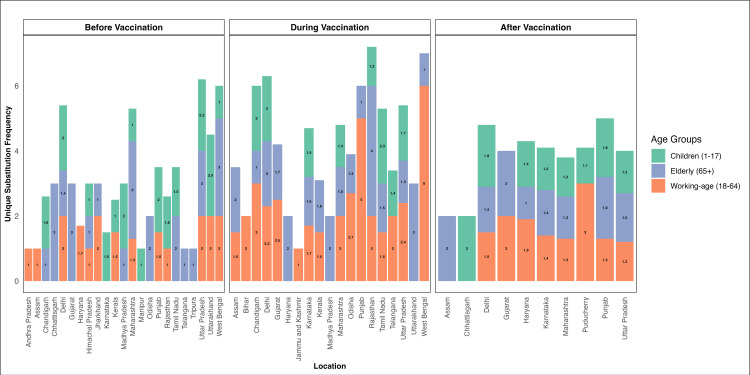
Dynamic changes in unique substitution frequencies influenced by the vaccination status and different states of India. The plot captures the profile vis-à-vis the different milestone of the COVID-19 footprint in India.

In Fig 8, the unique substitution frequency is presented to facilitate comparisons across multiple states and various conditions for different age groups. The data is divided into three age groups: children (1-17 years), elderly (65+ years), and working-age individuals (18-64 years), represented by green, blue, and orange bars, respectively. Unlike the plot for all substitutions, this plot focuses on unique substitutions, revealing distinct patterns. Before vaccination, unique substitution frequencies are relatively low across all age groups and locations. During vaccination, there is a noticeable increase in unique substitution frequencies, particularly in the working-age group, with some locations such as West Bengal and Panjab showing higher values. After vaccination, unique substitution frequencies decrease but remain notable in certain regions like Puducherry and Gujarat, especially among the working-age group.

We also performed the Friedman rank sum test for unique substitution frequencies, which also revealed significant differences across vaccination phases when stratified by age groups (χ² = 6, df = 2, p = 0.04979), highlighting temporal variations in unique substitution dynamics.

### The role of unique substitutions in shaping SARS-CoV-2 survival and infectivity

**Fig 9:**
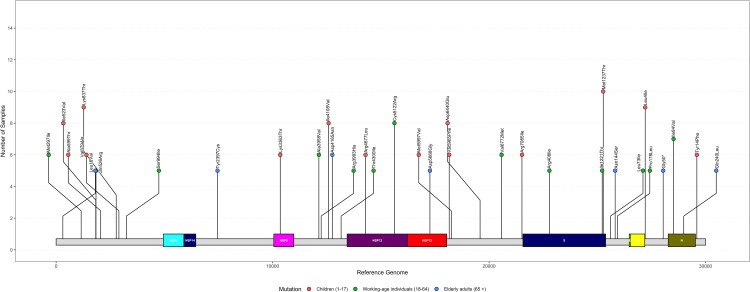
Substitution distribution plot across the SARS-CoV-2 genome, with substitutions categorized by age groups: Children (1–17), Working-age individuals (18–64), and Elderly adults (65+). Substitutions are plotted at specific positions on genes, with the Y-axis representing the number of samples in which unique substitution were found present.

We conducted an analysis to identify specific unique substitutions that were prevalent across specified age groups. To focus on biologically significant substitutions, we filtered the data by selecting only the top non-synonymous substitutions that were present in the maximum number of samples within each group (Children, working-age individuals, and elderly adults). This strategy allowed us to focus on mutations that were more likely to have biological relevance across the groups, making the analysis more manageable while retaining the most pertinent information. The Lollipop plot (Fig 9) shows only the top substitutions present in the maximum number of samples from each group.

In children aged 1 to 17, mainly in ORF1ab, ORF6, and Spike proteins substitutions were examined. Leu4Ile in ORF6 may modify non-structural protein domains, impacting viral replication fidelity [35]. Such substitutions allow viruses to optimize fitness in response to immune pressure [36]. Specifically, substitutions like Met1237Thr in the Spike protein might be involved in affecting viral entry by modifying the spike’s binding to the ACE2 receptor, which may enhance transmissibility or immune escape [37,38]. Research has shown that such Spike substitutions are critical for the evolution of SARS-CoV-2 variants, influencing vaccine resistance [39]. In children, who have developing or weaker immune systems, these adaptive changes are particularly relevant, underscoring how the virus co-evolves in response to host immunity pressures [40].

In the 18-64 age group, Key substitutions such as Met297Ile and Gln526Arg, identified in multiple samples, underscore the significance of these alterations within the cohort. Variants like Cys5122Arg in ORF1ab may affect viral replication efficiency and protein interactions [41,42]. Additionally, substitutions such as Arg408Ile in the Spike protein, which affects ACE2 receptor binding, can lead to increased viral transmissibility [43]. The emergence of variants like Delta (B.1.617.2) and Omicron (BA.2) with substitutions in the Spike and ORF1ab regions has demonstrated the virus’s ability to evade immunity, even in vaccinated populations [44,45].

Among elderly adults, notable substitutions were observed, predominantly in ORF1ab and ORF3a genes. Gly50*, a stop-gained substitution, and Asn144Ser were found in multiple samples. The detection of stop-gained substitutions like Gly50* in the ORF8 gene may lead to truncated protein functions, truncated ORF8 could affect antibody response, severity of infection and inflammatory response [46].

In addition to analysing individual substitutions, we evaluated substitution dynamics across specific genomic regions to provide a broader perspective. This was done by dividing the substitution count for each gene by its total nucleotide length, and the resulting values were then expressed as percentages. Table 4 summarizes the normalized synonymous and non-synonymous substitution rates across different genes for each group.

**Table 4:** Standardized synonymous and non-synonymous substitution counts across genes in different groups.

Gene	Synonymous Substitution Count			Non Synonymous Substitution Count		
	Group 1	Group 2	Group 3	Group 1	Group 2	Group 3
**E**	7.017	6.14	3.508	7.017	9.21	6.14
**M**	6.128	7.174	6.875	5.231	6.875	5.68
**N**	6.111	7.619	6.904	10.793	14.682	12.38
**ORF10**	5.982	6.837	6.837	18.803	21.367	29.059
**ORF1ab**	5.908	7.66	6.735	8.111	10.342	8.774
**ORF3a**	5.917	7.85	5.434	16.304	18.236	17.391
**ORF6**	5.494	8.791	10.989	14.835	13.736	13.186
**ORF7a**	6.4	6.666	6.933	12.266	16	13.866
**ORF7b**	5.303	6.818	6.06	9.848	12.121	14.393
**ORF8**	6.557	9.289	8.196	14.207	20.491	17.759
**S**	6.41	7.718	6.541	7.142	9.994	8.398

The data reveal varying substitution rates across genes, with ORF10 showing the highest non-synonymous substitution rates, particularly in the elderly group (Group 3), while E and M genes exhibit consistently lower substitution rates, indicating their conserved nature. Genes like N and S show moderate substitution rates, with an increase in Group 2 (working-age individuals) and Group 3 (elderly adults), suggesting age-specific pressures influencing mutation dynamics. This gene-specific breakdown highlights the variability in substitution patterns and provides a granular view of how substitutions shape viral evolution.

## Discussion

This study provides a comprehensive analysis of SARS-CoV-2 substitutions across different age groups in India, evaluating how these substitutions correlate with regional variations and vaccination phases. Our findings reveal a significant disparity in the number of unique substitutions, with working-age individuals exhibiting the highest count, followed by children and the elderly. This highlights a potentially higher substitution rate in the working-age population, which could be linked to increased exposure or differences in immune response. Notably, vaccination appears to influence substitution dynamics, with a marked increase in unique substitutions during the vaccination period across all age groups. This trend underscores the complex interplay between vaccination efforts and substitution rates, suggesting that while vaccination campaigns are crucial for controlling the virus, they may also contribute to heightened substitution diversity. The rise in unique substitutions during the vaccination period may be attributed to the virus evolving to survive in the presence of increasing population immunity. Vaccination exerts selective pressure on the virus, encouraging the emergence of substitutions that can evade the immune response [47]. This evolutionary process underscores the virus’s adaptability and survival mechanisms in response to widespread vaccination efforts. The lineage dynamics were also taken in account which revealed distinct patterns in the distribution of unique substitutions across different viral lineages and age groups. For lineages associated with the Delta variant, the elderly and pediatric groups exhibited a higher number of unique substitutions compared to the working-age group. In contrast, Omicron variants showed higher unique substitution counts in the working-age group. These findings suggest that different age groups may have unique immune responses that influence viral evolution. The analysis across different states reveals that working-age individuals (18-64 years) in India show a higher substitution uniqueness ratio (11.34%) compared to children (9.60%) and the elderly (9.22%). Significant regional variations exist, with states like Maharashtra and Karnataka exhibiting higher rates, while Kerala and Mizoram show lower percentages. These regional discrepancies may be attributed to factors such as variations in public health interventions, the presence of specific viral strains, or differences in transmission dynamics. Interestingly, while a study by Alsuwairi et al. [48] in Saudi Arabia reported a higher substitution frequency among elderly individuals, our research identified a greater prevalence of unique substitutions in the working-age adult cohort. This contrast highlights the variability of substitution patterns across different demographic groups and underscores the need for tailored surveillance strategies to address these unique regional and demographic differences. States like Maharashtra and Karnataka, which implemented rigorous testing and contact tracing, reported higher number of infected individuals. The emergence of transmissible variants, particularly the Delta variant, has been linked to significant surges in cases and substitutions, with specific strains dominating in certain regions, such as B.1.617.2 [49]. Additionally, local transmission dynamics influenced by population density and social behavior further contributed to these discrepancies [50]. This analysis included identification of unique SARS-CoV-2 substitutions across age groups, with a focus on those affecting protein structure. In children (1–17), substitutions in ORF6 and Spike proteins may influence viral replication and immune escape [35,37]. In working-age individuals (18–64), substitutions in ORF1ab and Spike, like Arg408Ile, were linked to increased transmissibility and immune evasion [42]. Among elderly adults (65+), stop-gained substitutions, such as Gly50*, were found, potentially affecting the immune response and infection severity [46]. These findings highlight age-related viral adaptations in response to host immunity. Research has demonstrated that young adults exhibit a notably high number of substitutions in SARS-CoV-2 strains, contributing to the virus’s rapid evolution and adaptation. A comprehensive analysis of over 10 million SARS-CoV-2 genome sequences revealed that certain substitutions in the spike protein, were prevalent, with substitution frequencies significantly higher in younger populations [51]. Furthermore, specific geographic regions have shown distinct substitution patterns, with certain substitutions occurring more frequently in Europe and North America [25].

Overall, understanding the evolution of SARS-CoV-2 is crucial as it is influenced by multiple factors, including host immune responses, geographic distribution, and viral replication mechanisms. For instance, SARS-CoV-2’s viral evolution has been extensively studied, highlighting the potential of translational research in advancing therapies, including antivirals, monoclonal antibodies, vaccines, and immunomodulators, to address both current and future challenges of COVID-19 [52]. Another study highlighted those collective non-synonymous substitutions in key proteins of SARS-CoV-2 showed a significant increase 10 to 14 days prior to rapid rises in cases, especially associated with variants like Gamma, Delta, and Omicron [53]. Hence, while the immediate crisis may have subsided, the need for vigilant surveillance is paramount to safeguard against potential future outbreaks and to adapt our healthcare strategies accordingly.

## Conclusion

This study offers a nuanced perspective on the substitution dynamics of SARS-CoV-2, highlighting the intricate interplay of factors such as age, geographical location, and vaccination status in India. By analyzing unique substitutions, it sheds light on how these variables collectively influence the virus’s evolution and spread, providing valuable insights. Notably, the finding that working-age group (18–64), generally known to exhibit stronger immune responses, showed the highest number of unique substitutions, plausibly suggests that heightened immune pressure may drive the virus to evolve more rapidly, resulting in a greater diversity of substitutions [54–56]. Moreover, the surge in unique substitutions observed in all age groups during the vaccination period reiterates the fact that enhanced immune responses, prompted by vaccination, might cause the virus to mutate at a higher frequency. Although the unique substitution counts decreased post vaccination, yet they remained elevated compared to pre-vaccination levels further supporting the idea that robust immune responses can lead to increased viral evolution. Although the COVID-19 pandemic is behind us, the findings of this study remain crucial for understanding the ongoing evolution of viral pathogens, especially single stranded RNA viruses. Based on our observations, virus surveillance can be strategically planned to account for differential effects across age groups. Specifically, individuals in the working-age group (18-64 years) may contribute to the development of more severe pathogenic mutations. This could be due to the higher transmissibility of the virus within this highly interactive/mobile demographic, combined with relative strong immune responses that may drive the evolution of viral traits. These factors likely accelerate the adaptation of SARS-CoV-2, enabling it to evolve in response to both natural and vaccine-induced immune pressures, leading to the emergence of variants with enhanced transmissibility or immune escape capabilities. To refine pandemic preparedness strategies, our findings suggest several key points for incorporation into surveillance policies. Targeted surveillance should focus on enhanced monitoring of working-age individuals due to their potential role in driving mutation dynamics. Adaptive vaccination strategies are necessary to adjust vaccination campaigns based on evolving viral traits, optimizing immunity and reducing breakthrough infections. Public health interventions should include region-specific containment measures to mitigate the spread of variants with increased transmissibility or immune escape potential. Viral evolution underlies how pathogens adapt to new environments, potentially gaining traits that enhance transmissibility, immune evasion, or resistance to treatment. By tracking substitutions and understanding their biological implications, we can predict how a virus may evolve and implement strategies to mitigate future risks. This knowledge is key for refining diagnostic tools and therapeutic interventions. In general, studying viral evolution enhances our ability to respond to emerging infectious diseases, offering insights into how pathogens may shift in response to human interventions, environmental changes, or host immune pressures. Such research supports not only public health strategies but also the broader field of disease prevention, ensuring that we are prepared to address future challenges posed by evolving viruses. Additionally, while this study specifically analyses the evolution of SARS-CoV-2 in India, its findings have broader implications for understanding viral evolution, especially single stranded RNA viruses, in other high-transmission countries such as the United States, and parts of Southeast Asia, that have faced similar challenges in controlling SARS-CoV-2 spread [57] and globally. The patterns of substitution and adaptation observed in the Indian population may provide valuable insights that are relevant to regions with similar socio-economic, healthcare, and demographic conditions [58]. A case in point is the new global regions, beyond the known cases within the tropical regions, wherein cases of Dengue virus infections are being reported in the recent years.

### Limitations to the Study

This study provides valuable insights into the evolution of SARS-CoV-2 across different age groups in India, yet, it has certain limitations that should be acknowledged to contextualize its findings. Firstly, the SARS-CoV-2 genome sequences were obtained from the GISAID database, which might not fully represent all geographic regions and demographics within India. Regions with more robust sequencing initiatives could be overrepresented, while others remain underrepresented due to limited resources. During incorporation of vaccination data, the lack of detailed individual-level data such as vaccine type and dose interval dates, may limit the accuracy of associations between mutations and vaccination status. While age is generally correlated with immune response, there might be exceptions among individuals within the same age groups, which has not been accounted. Similarly, the analysis of age-related variations in mutations did not account for comorbidities or other health conditions, which may confound the observed relationships. The analysis focuses exclusively on single nucleotide variations (SNVs), potentially overlooking other genomic alterations that could also play a crucial role in viral adaptation and pathogenicity. Lastly, while the study discusses the implications of its findings for vaccine development and public health strategies, practical implementation of these recommendations requires further interdisciplinary research integrating epidemiological, immunological, and social science perspectives.

## Supporting information

S1 File Accession Ids GISAID (gisaid.org https://search.app/rZoB74FRFaSaDmmRA)
(XLSX)
